# Conditioned pain modulation predicts persistent pain after knee replacement surgery

**DOI:** 10.1097/PR9.0000000000000910

**Published:** 2021-03-29

**Authors:** Christian Dürsteler, Yusmely Salazar, Uxia Rodriguez, Xavier Pelfort, Lluís Puig Verdié

**Affiliations:** a Pain Medicine Section, Anaesthesiology Department, Hospital Clínic de Barcelona, Barcelona, Spain; b Anaesthesiology and Critical Care Department, Kantonsspital Baden AG, Baden, Switzerland; c Anaesthesiology and Critical Care Department, Hospital del Mar, Barcelona, Spain; d Department of Orthopaedic Surgery, Consorci Sanitari de l'Anoia, Hospital de Igualada, Barcelona, Spain; e Department of Orthopaedic Surgery, Hospital del Mar, Barcelona, Spain

**Keywords:** Knee osteoarthritis, Persistent postsurgical pain, Hyperalgesia, Conditioned pain modulation

## Abstract

Persistent pain after total knee replacement is an underestimated outcome leading to significant health burden. Sensory testing has been explored to help surgeons in decision making and better patient selection. Patients with different chronic pain syndromes exhibit a poor descending pain inhibition that can be quantified through experimental paradigms (conditioned pain modulation). A poor preoperative descending pain inhibition response predicted persistence of pain after surgery in previous studies.

**Methods:**

This study investigated the correlation between a preoperative inefficient endogenous analgesia and a bad postoperative pain outcome (painful prosthesis). One hundred forty-six patients were studied preoperatively by quantitative sensory testing. Conditioned pain modulation was calculated as the relative decrease in pain intensity (thermal stimulus) during heterotopic painful stimulation.

**Results:**

Approximately 21.2% of patients had a bad pain outcome (painful prosthesis), 6 months after surgery. Preoperatively, 47.9% of patients exhibited an insufficient endogenous analgesia. The probability to develop persistent pain after surgery in that group was higher than that in patients with a sufficient endogenous analgesia (31.4% [20.9–43.6, 95% CI] vs 11.8% [5.5–21.3, 95% CI], respectively; *P* < 0.004). Correlation between conditioned pain modulation values and postoperative intensity of pain was also established. Besides, a preoperative lower quality of life (mental component) predicted a worse pain outcome, too.

**Conclusions:**

This cohort study shows that preoperative sensory testing predicts a bad pain outcome after total knee replacement. This tool could help clinicians in a better indication of patients with advanced knee osteoarthritis for replacement surgery.

**Registration Details:**

ClinicalTrials.gov: NCT01811888 (prospective).

## Abstract 2

Preoperative impaired endogenous analgesia correlates with chronic pain after knee replacement. Preoperative psychological distress is also related with this bad surgical outcome.

## 1. Introduction

Knee osteoarthritis is one of the leading causes of chronic pain and disability in developed countries. A 4-fold increase in its prevalence is anticipated by 2030 because of the progressive raising of its major risk factors (age and obesity).^7,20,23^ Pain (at rest, but particularly in movement) is the main symptom of knee osteoarthritis, and analgesic drugs, cardiovascular exercise, and weight loss are proposed as the most effective therapeutic strategies.^8^ Unfortunately, this approach has a modest impact on patients' pain, function, and quality of life.^6,15^ Knee replacement (KR) is indicated in patients with pain, which is refractory to conservative analgesic therapy and severe joint damage.^22^ It is a cost-effective procedure and one of the most frequent in-patient surgeries, but its economic burden is enormous.

Chronic pain after KR has not been adequately quantified. Painful prosthesis is defined by the community of orthopaedic surgeons as the persistence of pain in the operated knee after replacement surgery, without any evidence of infection, loosening of components, or knee misalignment.^12^ The few published reports about this subject show various incidences of persistent pain after KR surgery (20%–40%), and their definition is rather vague for time of diagnosis, pain assessment (rest vs movement), and impact on function and quality of life.^21^

Persistent pain after surgery has been increasingly studied since first reported in the literature in 1998.^4^ Its physiopathology has not yet been elucidated, but preoperative (pain and anxiety), intraoperative (nerve damage), and postoperative (intense pain) risk factors have been proposed. The identification of consistent perioperative clinical risk factors allows us to develop risk scores to inform our patients of their probability of developing persistent postoperative pain^3,23^ before some types of surgery.

In parallel, some research groups have focused their efforts on the study of the relationship between the efficacy of descending inhibitory systems (or more precisely, the final balance between the descending inhibitory and facilitatory drive, the so-called pain modulation profile) and the development of persistent pain after surgery. In this way, a poor result in the conditioned pain modulation (CPM) experimental protocol (pain inhibition by heterotopic painful stimulation) or the presence of temporal summation (windup phenomenon) showed significant predictive power.^17,29^ Thus, psychophysical evaluation (sensory testing) has been advocated as a valuable tool in persistent postsurgical pain research and the integration of clinical and experimental data recommended for future quality studies.^13^

Preoperative identification of patients at risk of developing persistent pain after surgery could allow us to focus research on this patients group and implement (future) preventive strategies only in the susceptible population. The objective of this study is to investigate whether preoperative CPM can predict persistent pain after KR surgery.

## 2. Methods

### 2.1. Protocol

From August 2014 to February 2017, 180 patients scheduled for primary total knee arthroplasty were recruited consecutively by the orthopaedic surgery department. Patients were aged older than 18 years. Important exclusion criteria were treatment with antidepressants in the past 3 months, previous surgery on the target knee, and documented peripheral neuropathy. These conditions (previous surgery and neuropathy) could interfere with CPM, and antidepressant drugs are known modulators of descending pain inhibitory systems. A complete list of inclusion and exclusion criteria can be found at http://www.clinicaltrials.gov (NCT01811888).

All patients gave their written informed consent before enrolment. The protocol fulfilled the requirements of the Spanish Organic Law 15/1999 of December 13, 1999, which regulates the protection of personal data, and was approved by our institution's ethics committee (2013/4728/I). The study was undertaken in accordance with the Declaration of Helsinki and performed according to the principles of the International Conference on Harmonisation Guidelines for Good Clinical Practice (1996 revision) in the European Community.

### 2.2. Study design and data collection

This research project was a cohort study, and the Strengthening the Reporting of Observational studies in Epidemiology (STROBE) guidelines were used to ensure appropriate reporting.^26^ The list of patients scheduled for KR was facilitated by the orthopaedic surgery department on a monthly basis. Patients on the list were consecutively contacted by phone to be invited to participate in the study. A first visit (baseline) was arranged to thoroughly inform them about the clinical and experimental aspects of the study. After agreement and informed consent signature, patient-reported outcomes as well as clinical characteristics of patients were provided by patients on case report forms in a quiet office. Afterwards, the experimental CPM protocol was performed in a silent and temperature-controlled (23 ± 1°C) room. All experimental procedures were performed during the week before surgery by a single examiner (Y.S.).

Patients were seen at scheduled study visits at baseline and after 6 months. Between the visits, patients were contacted by phone at 3 months to register their pain scores (numerical rating scale [NRS], at rest and in movement).

The primary outcome was the proportion of patients with persistent pain (NRS >3 at rest) 6 months after surgery. The secondary outcomes included pain scores at rest and in movement (week 1, month 3, and month 6) and Western Ontario and McMaster Universities Osteoarthritis Index (WOMAC 3.1), Neuropathic Pain Symptom Inventory (NPSI), Hospital Anxiety and Depression Scale (HADS), and SF-36 quality of life questionnaire (all questionnaires were self-administered at week 1 and month 6).

### 2.3. Preoperative experimental protocol: conditioned pain modulation

Conditioned pain modulation was calculated as the negative difference in pain rating between 2 identical noxious “test stimuli” applied first at baseline and then concomitantly with another “conditioning” remote noxious stimulus, with the following formula:

$$\text{CPM}=-\left({\text{NRS}}_{\text{post}}-{\text{NRS}}_{\text{pre}}\right).$$

A decrease in the “test stimulus” pain score from the baseline indicates efficient pain inhibition, expressed as a positive CPM value. The “test” stimulus was a 30-second contact heat stimulus (TSA-II NeuroSensory Analyzer, Medoc, Israel) applied to the volar aspect of the dominant forearm. The intensity of this stimulus was previously estimated for each individual using a short series of ascending and descending thermal stimuli, culminating in the identification of the temperature that induced a pain score of 6 on a 0 to 10 NRS (for more details, see Granot et al.^9^). The pain magnitude of the 30-second “test stimulus” was the mean of 3 stimuli, administered every 10 seconds during stimulation.

The “conditioning stimulus” was delivered by immersion of the other hand in a hot water bath (46.5°C) with a stirring system (Agibat 20, JP Selecta, Spain) for 1 minute. A second assessment of the “test stimulus” was performed during the last 30 seconds of the immersion time, and patients were asked to report pain magnitude 3 times (after 40, 50, and 60 seconds) during this repeated test stimulus.

### 2.4. Surgical procedure, postoperative pain management, and rehabilitation

Total knee arthroplasties were performed by 4 different expert orthopaedic surgeons using the same technique, components (Triathlon; Stryker, Kalamazoo, MI), and cement (Simplex P bone cement; Stryker). All patients received prophylactic intravenous antibiotic (2 g of cefazolin or 1 g of vancomycin in penicillin-allergic patients) and were operated on under tourniquet at a pressure of 350 mm Hg with previous exsanguination. A midline skin incision and a medial parapatellar capsulotomy were made.

All surgeries were performed under spinal anaesthesia with hyperbaric 0.5% bupivacaine (10–15 mg), and patients received a routine intravenous postoperative pain management protocol, consisting of 1 g of paracetamol/6 hours, 50 mg of dexketoprofen/8 hours, 100 mg of tramadol/6 hours, and a femoral and sciatic nerve block (30 + 20 mL of 0.25% bupivacaine, respectively). Patients received subcutaneous rescue morphine (0.1 mg·kg^−1^) every 6 hours, when needed.

From day 1 postoperatively, all patients received 3 weeks of rehabilitation, improvement mainly consisting of motion of the knee; manual movement of the knee by the therapist; isometric quadriceps exercises and assisted exercises (active knee flexion and extension and progressive muscle strengthening ceremonies); gait training and transfer; and training on stairs, ramps, and obstacles. At 3 weeks, patients who did not achieve 90° of flexion or had a total length deficit continued outpatient therapy; patients who achieved a satisfactory range of motion were discharged with instructions to perform their exercises at home.

### 2.5. Statistical analysis

A study size of 180 patients was chosen based on the assumption of the presence of insufficient endogenous analgesia in 30% of the general population (described in previous studies^27^) and an estimated 15% dropout ratio. We accepted an alpha risk of 0.05 and a beta risk of 0.2 in a unilateral contrast, assuming a persistent pain after a surgery rate of 44% (highest persistent pain rate described in previous studies) for patients with insufficient endogenous analgesia and a reduction of up to 20% for patients with efficient endogenous analgesia.

Data are presented as means and standard deviation, unless otherwise stated. Comparative analysis was made with an independent sample *t* test for continuous variables or its nonparametric equivalent Mann–Whitney *U* (Wilcoxon) test, depending on the inherent characteristics of the studied variables. Comparisons of categorical variables were made using the Pearson χ^2^ test. Comparisons between preoperative and postoperative SF-36 and WOMAC 3.1 questionnaire scores were made with the paired *t* test or Wilcoxon signed-rank sum test in case of nonnormal data distribution.

Correlations were calculated using the Pearson correlation coefficient (*r*) as a measure of the strength of the association between the variables studied. If a normal distribution of data was demonstrated, the *t* test was used to establish whether the correlation coefficient was significantly different from zero, suggesting an association between the 2 variables. Data management and statistical analysis were performed using SPSS v.18.0 (IBM Corp, Armonk, NY).

## 3. Results

**Figure 1.**
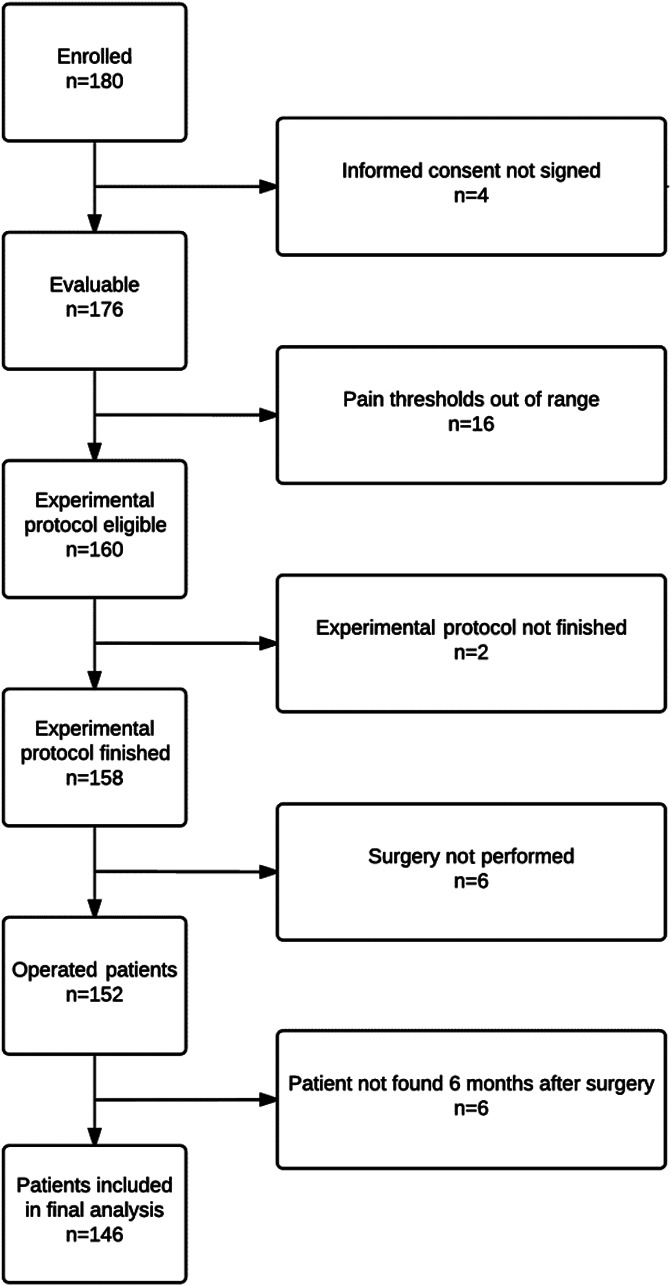
Flowchart indicating selection of study participants.

Approximately 97.8% of the 180 patients invited to participate met all the inclusion and none of the exclusion criteria and signed the informed consent form (4 of them refused to participate in the CPM protocol and did not). Of the 176 evaluable patients, the total missing (30 patients) were attributable to different reasons: surgery finally not performed (6), lost to follow-up 6 months after surgery (6), early termination of experimental protocol (2), and experimental protocol not commenced because of out-of-range pain thresholds (16). Figure 1 shows a flowchart of the study. Results of this research work can be classified into clinical and experimental results.

### 3.1. Clinical results

#### 3.1.1. Demographic characteristics

The mean age (and SD) of the 146 patients who completed the study was 73.1 (7.1) years and 73.3% of them were women, with a mean body mass index of 31.2 (5.4) kg/m^2^.

#### 3.1.2. Pain data

Mean preoperative pain (and SD) intensity (target knee) measured with an NRS was 3.6 (3.2) at rest and 7.9 (2.0) in movement (15 minutes walking). 50.7% (42.3–59.1, 95% CI) of patients and 97.9% (94.1–99.6, 95% CI) of patients had a pain score more than 3 (NRS > 3) at rest and in movement, respectively, as summarized in Table 1. Measures of pain, stiffness, and physical function were also registered using the WOMAC 3.1, as summarized in Table 1. Regarding the psychometric characteristics of knee pain, postoperative NPSI questionnaire scores were significantly lower compared with the preoperative scores for global (10.08 [13.53] vs 27.01 [17.16], respectively) and for each dimension of NPSI.

**Table 1:** Clinical variables registered before and after knee replacement surgery.

	Preoperative (week 1)	Postoperative		*P*
		3 mo	6 mo	
Pain intensity (target knee)				
At rest, NRS, mean (SD)	3.6 (3.2)	2.6 (2.3)	1.8 (2.3)	0.012
In movement, NRS, mean (SD)	7.9 (2.0)	4.0 (2.4)	4.2 (2.7)	0.001
Presence of NRS >3 (target knee)				
At rest, n (%)	74 (50.7)	46 (31.7)	31 (21.2)	
In movement, n (%)	143 (97.9)	87 (60.0)	86 (58.9)	
Pain in other locations (low back, contralateral knee, and shoulder, the most frequent painful areas, other than target knee), n (%)	93 (63.79)			
Pain intensity (other locations)				
At rest, NRS, mean (SD)	4.2 (3.3)			
In movement, NRS, mean (SD)	7.1 (2.5)			
Presence of NRS >3 (other locations)				
At rest, n (%)	55 (59.1)			
In movement, n (%)	84 (90.3)			
NPSI, mean (SD)				
Burning pain	2.64 (3.72)		1.83 (2.90)	0.035
Paresthesia/dysesthesia	3.20 (2.72)		1.21 (1.86)	0.001
Paroxysmal pain	2.90 (3.20)		1.07 (2.08)	0.001
Pressing pain	4.83 (3.04)		1.85 (2.43)	0.001
Evoked pain	1.80 (2.12)		0.72 (1.25)	0.001
Total score	27.01 (17.16)		10.08 (13.53)	0.001
WOMAC 3.1				
Pain, mean (SD)	10.3 (3.3)		5.5 (3.6)	0.001
Stiffness, mean (SD)	3.5 (2.1)		2.0 (1.7)	0.001
Physical function, mean (SD)	34.7 (11.4)		18.27 (12.37)	0.001
HADS				
Anxiety score >8, n (%)	41 (28.5)		38 (17.6)	0.075
Depression score > 8, n (%)	21 (14.5)		19 (13.2)	0.591
SF-36				
Physical component score, mean (SD)	31.3 (6.7)		39.6 (8.2)	0.001
Mental component score, mean (SD)	41.0 (14.4)		48.7 (12.7)	0.001

NPSI score range: 0 to 5 (no symptom to worst symptom); WOMAC 3.1 score range: pain = 0 to 20 (no pain to worst), stiffness = 0 to 8 (no stiffness to worst), and physical function = 0 to 68 (best function to worst); HADS: A total subscale score of >8 points of a possible 21 denotes considerable symptoms of anxiety or depression; NRS: 0 to 10 (no pain to worst pain).

HADS, Hospital Anxiety and Depression Scale; NRS, numerical rating scale; NPSI, Neuropathic Pain Symptom Inventory.

A broader pain topographic investigation showed that 63.7% (55.3–71.5, 95% CI) of patients had pain in other locations, with low back pain (with and without leg radiation) and contralateral knee and shoulder pain being the most common. Mean pain intensity in these other body parts was similar to that obtained in the studied knees (Table 1).

Statistically significant differences in preoperative pain scores (target knee) were observed between women and men, detecting higher pain levels at rest and in movement in women (*P* < 0.006 and *P* < 0.005, respectively) compared with men (Table 2). A higher proportion of women showed a preoperative pain score level more than 3 (NRS, target knee) at rest (*P* < 0.001), doubling the proportion found in men. Higher preoperative pain levels in other locations (in movement) were also found in women compared with those in men (*P* < 0.019), as well as higher preoperative anxiety level (HADS score >8; *P* < 0.011).

**Table 2:** Clinical and experimental sex differences in advanced knee osteoarthritis patients for knee replacement.

	Female	Male	*P*
Frequency, n (%)	107 (73.3)	39 (26.7)	
Preoperative pain intensity, target knee, NRS, mean (SD)			
At rest	4.0 (3.2)	2.4 (2.9)	0.006
In movement	8.2 (1.9)	7.1 (2.2)	0.005
Preoperative NRS > 3, target knee, NRS, mean (SD)			
At rest	63 (58.9)	11 (28.2)	0.001
In movement	63 (58.9)	105 (98.1)	1
Preoperative pain intensity in other locations (low back, contralateral knee, and shoulder, the most frequent painful areas, other than target knee), NRS, mean (SD)			
At rest	4.5 (3.3)	3.1 (3.0)	0.108
In movement	7.4 (2.5)	5.9 (2.1)	0.019
Preoperative anxiety, HADS score >8, n (%)	36 (34.3)	5 (12.8)	0.011
Preoperative depression, HADS score> 8, n (%)	19 (17.9)	2 (5.1)	0.052
Preoperative CPM score, mean (SD)	0.43 (1.66)	0.26 (2.21)	0.649
Preoperative presence of insufficient endogenous analgesia, n (%)	49 (45.8)	21 (53.8)	0.389

CPM, conditioned pain modulation; HADS, Hospital Anxiety and Depression Scale; NRS, numerical rating scale.

Regarding preoperative pain therapy, 65.8% (57.5–73.4, 95% CI) of patients were treated with paracetamol + nonsteroidal anti- inflammatory drugs (NSAIDs), 13.7% (8.6–20.4, 95% CI) with a combination of paracetamol + NSAIDs + tramadol, and only 6.2% (2.9–11.4, 95% CI) with strong opioids added to their treatment (paracetamol + NSAIDs + strong opioids). Surprisingly, 14.4% (9.1–21.1, 95% CI) of patients received no analgesic treatment at all.

##### 3.1.2.1. Persistence of pain after surgery

The proportion of patients with persistent pain at rest (NRS > 3) at 3 months after surgery was 31.7% (24.3–40, 95% CI), whereas the incidence of persistence of pain at rest at 6 months after surgery (our primary outcome) was 21.2% (14.9–28.8, 95% CI). This decrease in pain at rest observed 3 months later could not be demonstrated for pain in movement (15-minute walk; 60% [51.5–68, 95% CI] of patients vs 58.9% [50.5–67, 95% CI]) at 3 and 6 months after surgery, respectively).

#### 3.1.3. Quality of life

Postoperative SF-36 physical and mental component summary scores (39.6 [8.2] and 48.7 [12.7], respectively) were significantly higher (*P* < 0.001) compared with preoperative scores (31.3 [6.7] and 41.0 [14.4], respectively), demonstrating a global favourable outcome for KR surgery for quality of life (Table 1). Patients with persistent pain after KR showed lower scores in the SF-36 (physical and mental components) compared with scores of patients without persistent pain (35.93 [7.34] vs 40.66 [8.06] for physical component, *P* < 0.011; and 40.50 [12.86] vs 51.02 [11.91], *P* < 0.001 for mental component, respectively), as summarized in Table 3.

**Table 3:** Differential clinical and experimental characteristics in patients with persistent postsurgical pain after knee replacement compared with patients with a favourable pain outcome.

	Non-PPP	PPP	*P*
Frequency, 6 mo after surgery, n (%)	115 (78.8)	31 (21.2)	
Preoperative efficient endogenous analgesia, n (%)	67 (88.2)	9 (11.8)	
Preoperative insufficient endogenous analgesia, n (%)	48 (68.6)	22 (31.4)	0.004
Preoperative NPSI			
Burning pain	2.26 (3.51)	3.77 (4.10)	0.068
Paresthesia/dysesthesia	2.98 (2.65)	4.15 (2.77)	0.034
Paroxysmal pain	2.83 (3.09)	4.08 (3.23)	0.049
Pressing pain	4.23 (3.13)	5.63 (2.77)	0.025
Evoked pain	1.71 (1.92)	2.49 (2.54)	0.067
Total score	25.22 (15.84)	34.60 (18.29)	0.006
Preoperative WOMAC 3.1			
Pain, mean (SD)	5.12 (3.43)	5.91 (3.82)	0.266
Stiffness, mean (SD)	1.92 (1.49)	2.23 (1.80)	0.883
Physical function, mean (SD)	18.48 (11.88)	18.07 (12.91)	0.835
Preoperative HADS			
Anxiety score >8, n (%)	51 (49.5)	52 (50.5)	0.328
Depression score> 8, n (%)	75 (51.7)	70 (48.3)	0.591
Preoperative SF-36			
Physical component score, mean (SD)	30.88 (6.60)	33.04 (6.93)	0.112
Mental component score, mean (SD)	42.48 (14.58)	35.69 (12.55)	0.019
Postoperative SF-36			
Physical component score, mean (SD)	40.66 (8.06)	35.93 (7.34)	0.011
Mental component score, mean (SD)	51.02 (11.91)	40.50 (12.86)	0.001

PPP: persistent postsurgical pain after knee replacement surgery (NRS > 3 at rest, 6 months after surgery); NPSI score range: 0 to 5 (no symptom to worst symptom); WOMAC 3.1 score range: pain = 0 to 20 (no pain to worst), stiffness = 0 to 8, (no stiffness to worst), and physical function = 0 to 68 (best function to worst); HADS: A total subscale score of >8 points of a possible 21 denotes considerable symptoms of anxiety or depression.

HADS, Hospital Anxiety and Depression Scale; NPSI, Neuropathic Pain Symptom Inventory.

#### 3.1.4. Physical function

Physical function improved significantly 6 months after surgery when comparing preoperative and postoperative results of WOMAC 3.1. questionnaire, as well as stiffness and pain, as summarized in Table 1.

#### 3.1.5. Anxiety and depression

Women exhibited a higher preoperative incidence of anxiety compared with men (34.3% [25.3–44.2, 95% CI] vs 12.8% [4.3–27.4, 95% CI], respectively; *P* < 0.011). Higher incidences of preoperative depression were also observed in women compared with those in men (17.9% [11.2–26.6, 95% CI] vs 5.1% [0.6–17.3, 95% CI]), but the difference was not statistically significant (*P* < 0.052), as summarized in Table 2.

### 3.2. Experimental data

**Figure 2.**
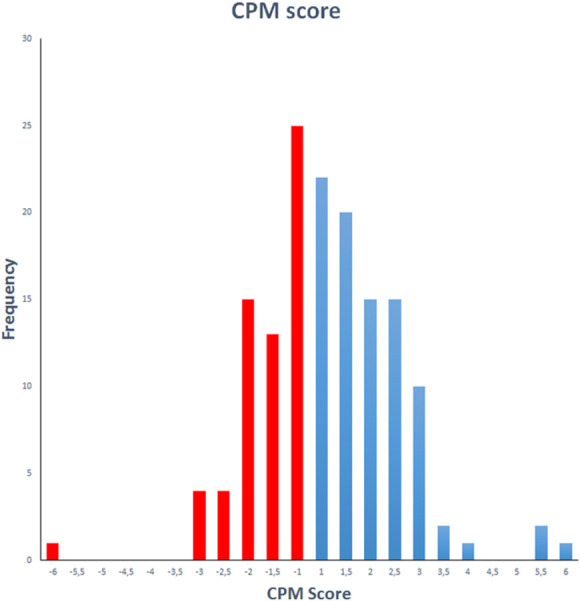
Histogram of CPM values. Mean (SD) = 0.39 (1.82). N = 146. Red bars show patients with preoperative insufficient endogenous analgesia. CPM, conditioned pain modulation.

The mean (SD) of CPM value was 0.39 (1.82), with a range of −6 to +6. A Kolmogorov–Smirnov test showed a *P* value of 0.592, indicating a normal distribution of this variable (Fig. 2).

Patients with insufficient endogenous analgesia (poor pain inhibition) had CPM values equal or less than 0, whereas patients with positive CPM values were considered to have an efficient endogenous analgesia (strong pain inhibitors). Approximately 47.9% (39.6–56.4, 95% CI) of patients showed insufficient endogenous analgesia before surgery.

No association was found between CPM values and any other variables measured in our study (sex, age, preoperative pain intensity [in the target knee and other locations], anxiety and depression, WOMAC 3.1 scores, NPSI scores, SF-36, etc.).

### 3.3. Correlations

No differences in pain persistence were observed between patients with insufficient endogenous analgesia and those with efficient endogenous analgesia 3 months after surgery. In addition, no early correlation was found between preoperative CPM values and postoperative intensities of pain (at rest or in movement) on the operated knee. However, a correlation was found between preoperative insufficiency of endogenous analgesia and the presence of persistent pain 6 months after surgery, *P* < 0.004 (Table 3). Preoperative insufficiency of endogenous analgesia also showed a good correlation with pain intensity at rest (not in movement). Thus, patients with insufficient preoperative endogenous analgesia had a higher pain intensity at rest 6 months after surgery (NRS, mean [SD]: 2.3 [2.6]) compared with patients with efficient preoperative endogenous analgesia (1.2 [2.0], *P* < 0.006).

A statistically significant correlation (*P* < 0.038) was also found between preoperative CPM values and pain intensity at rest (not in movement) 6 months after surgery. Thus, patients with lower preoperative CPM values (poor pain inhibitors) exhibited a higher pain level compared with patients with higher preoperative CPM values (strong pain inhibitors).

## 4. Discussion

This study showed that preoperative CPM is able to predict persistent pain after KR surgery in patients with advanced osteoarthritis. Patients with a preoperative low score in CPM protocol (poor pain inhibitors) exhibited a 2-fold increase in the incidence of persistent pain 6 months after surgery. In addition, the only nonexperimental preoperative variable associated with persistence of pain was a low score in the mental component of SF-36 quality of life questionnaire.

Conditioned pain modulation protocol demonstrated that nearly half of the patients (47.9%) had a poor preoperative pain modulation profile (insufficient endogenous analgesia). These results are consistent with previous studies in osteoarthritis, which propose CPM as a robust tool for a large age range and reliable for long-term follow-up studies.^16^

Previous works demonstrated alterations in the pain modulatory profile after KR surgery, with low CPM values in patients with persisting pain and restoration of CPM function in patients without persisting pain.^10,24^ Regarding the ability of preoperative CPM to predict persistent pain after KR surgery, a Danish group revealed no association for CPM values but a good correlation for temporal summation.^17^ Later on, they demonstrated that a patient group with a high preoperative pain facilitation to inhibition ratio exhibited less pain relief after surgery.^18^ A more recent study showed lower pain scores after total KR in patients exhibiting hypoalgesia after a cold pressor test and aerobic exercise.^25^

Discrepancies between our findings and those of previous studies could be attributed to differences in the type of conditioning and test stimuli used, as well as the outcome measurement methods used. Results obtained using the cold pressor test as a CPM paradigm may be different from using heat pain as a test stimulus and a hot immersion bath as a conditioning stimulus. Unfortunately, these discrepancies are difficult to explain, and the best CPM protocol is not yet established. Test and retest reliability of the CPM protocol has been proved in a review,^14^ but comparison of values obtained in the same population with different CPM protocols (different conditioning and test stimuli used) has been inadequately studied. A recent study showed that intersession reliability was robust for a diverse test and conditioning stimuli (pressure pain threshold and cold pressor test as a test stimulus and conditioning stimulus, respectively, are the most reliable^12^). No significant CPM effect was found in the electrical and heat pain thresholds with the cuff conditioning stimulus in contrast to the other pain detection thresholds, suggesting the possibility that the CPM phenomenon could be divided into deep-somatic conditioning stimulus-driven and cutaneous conditioning stimulus-driven circuits, which calls for deeper insights into future studies. Thus, different results when different tools and outcome measures are used cannot be dismissed because they would account for the differences obtained in similar populations studied with different tools.

Another interesting finding of our work is the presence of a lower preoperative mental component of the SF-36 quality of life questionnaire in patient group exhibiting persistent pain after surgery. Not only clinical but also preclinical studies have consistently identified psychological preoperative risk factors of chronic postsurgical pain, including negative affective constructs, anxiety symptoms, depressive symptoms, pain catastrophizing, and general psychological distress.^2,19,28^ However, another study did not find any association between the preoperative pain catastrophizing scale and pain intensity 1 year after surgery.^11^ Anxiety and depression are common preoperative features in surgical population. In our study, female patients (73.3%) exhibited anxiety and depression (34.3% and 17.9%, respectively); surprisingly, a specific questionnaire for anxiety and depression (HADS) was not able to predict persistent postsurgical pain.

Knee replacement surgery will be one of the most frequently performed surgical interventions in the near future. Estimations of rates of total KR in European (and other) countries are alarmingly high, and the economic implications will require specific health policies (obesity control in adults, but particularly in children).^1,5^ Assuming that nearly 20% of patients will develop persistent postsurgical pain (painful knee arthroplasty), the search for tools addressed to identify the group at risk of developing persistent pain after surgery becomes more important. The osteoarthritis population has been widely studied (psychophysical tests), but most of the published works are still lacking fusion between experimental and clinical variables. This study aimed to contribute to this research field not only with the CPM paradigm but also associating clinical preoperative and postoperative relevant parameters (pain at rest and in movement, quality of life, anxiety and depression, and physical status, among others). Considering CPM alone to help orthopaedic surgeons with decision making will most likely be a reductionist approach; combining CPM with predictive clinical-based algorithms could be the best strategy. After the identification of the best tool to classify KR candidates in different risk groups, a perioperative preventive intervention could be designed to be used only in the high-risk group, avoiding to treat the whole surgical population. Future advances in physiopathology of pain modulation knowledge and its relationship with specific chronic pain syndromes should be based on studies that combine clinical predictive tools with experimental CPM protocols.

## Disclosures

The authors have no conflicts of interest to declare.
